# The Case of the Noisy Neurons

**DOI:** 10.1371/journal.pbio.0020314

**Published:** 2004-08-24

People are unpredictable. One night you may crave Italian food, but another only Thai will do. One day you might finish a crossword puzzle in record time, and the next not a single clue prompts an answer. Such behavioral variation has been found in laboratory studies, too: a person's ability to find a faint image on a screen varies widely from one viewing to the next. Similarly, when an animal repeatedly receives the same stimulus—for example, a faint image—a neuron in a region of the animal's visual brain might be very active upon one presentation and relatively quiet the next.

Across the cerebral cortex—the brain region that integrates the senses and controls voluntary movement—neurons are notorious for their unpredictable behavior. The neurons themselves don't create this noise; when directly stimulated with an electrode multiple times, neurons will give the same response every time. Most neurons, however, receive signals from a host of other neurons. These various signals combine to form a seemingly noisy electrical input, which shows up as fluctuations in the recipient neuron's membrane potential—a difference in electrical charge between the inside and outside of the cell's membrane. Neuron function is intimately tied to the membrane potential, which is usually maintained within a narrow range, called the resting potential. But incoming signals can push the resting potential higher or lower. If the membrane potential rises above a certain threshold, the neuron fires, sending an electrical signal down its length. In this way, the brain relays and processes information.

Since the 1960s, neuroscientists trying to account for the cortex's variable responses have pointed to noisy inputs from other parts of the brain as the prime suspect. In this issue of *PLoS Biology*, Matteo Carandini addresses this longstanding mystery of neuron variability and comes up with a different answer. Carandini simultaneously measured the membrane potentials and firing patterns of individual neurons in the cat visual cortex. He found, surprisingly, that the membrane potentials varied much less than the firing patterns, ruling out noisy inputs as the cause of neurons' noisy outputs. Instead, the neurons amplified noise in the signals they received.[Fig pbio-0020314-g001]

**Figure pbio-0020314-g001:**
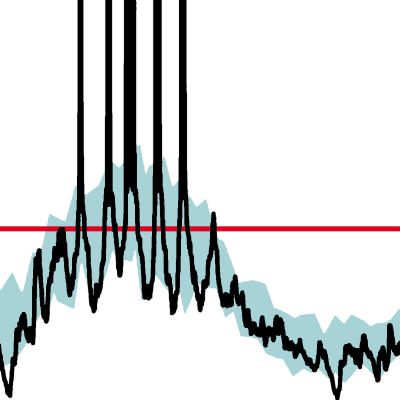
Noise and threshold make neurons unpredictable

Carandini then used a simple model of neuron behavior to explain why this would occur. He started with a tried-and-true approximation of neuron behavior, called the rectification model: a neuron doesn't fire until its membrane potential rises above a threshold, but once it crosses this threshold, its firing rate is correlated with the strength of incoming signals. Then he added the assumption that the neurons receive signals with some randomness. Given these minimal assumptions, Carandini showed that neurons fed a noisy signal will tend to amplify the noise in the signal. Importantly, his model reproduced a well-known phenomenon: as cortical neurons' average firing rate goes up, their firing rate also becomes more variable—that is, they get noisier.

Carandini's model also predicted something new: as the firing rate continues to increase, the firing rate should become more consistent and less noisy—which he calls saturation of variability. Carandini's measurements in cats showed neurons actually behave this way, a key validation of his model.

It's not clear whether this amplification of variability is something that helps or hampers the brain. Despite being a nuisance to neuroscientists, such fluctuations could be crucial to how the brain functions, Carandini speculates. Without some variability in their cortex, animals would act like cameras or other simple machines that respond the same way each time to a stimulus. It's advantageous for behavior, and hence brains, to be adaptable. But amplifying noise in a signal seems to run counter to relaying and processing the information in the signal. Carandini suggests that what appears as noise in the experiments are signals from other parts of the cortex—that is, noise is in the eye of the beholder. Now that the source of the variability is clear, neuroscientists can study whether it serves a function in the brain.

